# Rare complication of hepatocellular carcinoma in Wilson's disease

**DOI:** 10.1002/jgh3.12648

**Published:** 2021-08-27

**Authors:** Marina Ohkoshi‐Yamada, Kenya Kamimura, Hiroteru Kamimura, Shuji Terai

**Affiliations:** ^1^ Division of Gastroenterology and Hepatology Graduate School of Medical and Dental Sciences, Niigata University Niigata Japan; ^2^ Department of General Medicine Niigata University School of Medicine Niigata Japan

**Keywords:** D‐penicillamine, fatty infiltration, hepatocellular carcinoma, Wilson's disease

## Abstract

The complication of hepatocellular carcinoma (HCC) in Wilson's disease is rare. Wilson's disease treatment using D‐penicillamine (DPA) is useful to prevent HCC occurrence; however, it also causes iron accumulation and synergistic radical formation in the liver, which may enhance carcinogenesis. Reported herein is a case of HCC in Wilson's disease treated with DPA for 36 years. The tumor was surgically resected and histologically diagnosed with moderately differentiated HCC surrounded by cirrhotic tissue with fatty infiltration. Rhodanine staining revealed a slight positively stained area in both tumor and surrounding tissues. Information obtained from this case and literature review highlight the feature of HCC in Wilson's disease.

## Graphical abstract

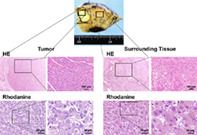

A complication of the hepatocellular carcinoma (HCC) in Wilson's disease is rare. The treatment for Wilson's disease using D‐penicillamine is useful to prevent HCC occurrence; however, it also causes iron accumulation and synergistic radical formation in the liver which may enhance the carcinogenesis. We report a case of HCC in Wilson's disease treated with D‐penicillamine for 36 years.

## Introduction

Wilson's disease is an autosomal recessive genetic disorder, and *ATP7B*, the responsible gene that is located on chromosome 13, has mutated, resulting in functional failure of ATP7B protein as copper‐transporting protein. This results in impaired copper transportation from the cytosol to the Golgi apparatus and excretion into bile.^1^ Copper accumulation in the cytosol and reduced holo‐ceruloplasmin synthesis result in increased unbound copper in blood that causes organ failure due to copper accumulation in multiple organs.^1^ This copper deposition leads to various pathologies depending on involved organs, including Kayser–Fleischer rings, general malaise, jaundice, acute liver failure, chronic liver failure, liver cirrhosis, liver cancer, dysarthria, sardonic grin, tremors, salivation, character changes, renal disorders, joint problems, endocrinopathy, heart failure, and arrhythmias.^1^, ^2^, ^3^ Disease incidence is approximately one in 40 000 people, with estimated one carrier for every 100 people,^4^ and early diagnosis of the disease is difficult. The peak onset is approximately 10–11 years of age; however, age of onset can vary widely from 3 to 50 years.^5^ Additionally, risk of primary liver cancer development in patients with Wilson's disease is reported to be low,^6^ and a retrospective study of 1186 patients with Wilson's disease in Europe found a 1.2% prevalence of liver cancer with an incidence of 0.28 cases per 1000 person years. A previous report noted that among 45 cases of liver cancer in patients with Wilson's disease, where the degree of hepatic fibrosis was clearly known, the onset of cancer started with liver cirrhosis in 41 cases.^7^ In another retrospective study of 130 patients with Wilson's disease, only two cases (1.5%) were hepatocellular carcinoma (HCC), with HCC occurrence rate of 0.09% from noncirrhotic liver and 0.14% from cirrhotic liver during the median observation period of 15 years.^8^, ^9^, ^10^ Information regarding images and clinical course of HCC in Wilson's disease was not minutely reported to date due to rarity. Reported herein is a case of HCC complicated with Wilson's disease, which was treated with D‐penicillamine (DPA) for 36 years. Results support the importance of long‐term follow‐up even under good management of various symptoms. DPA is a dimethylated cysteine and a key copper chelator, which promotes excess copper excretion into the urine.^1^, ^2^, ^11^ Effect on neurological symptoms is questionable as it may increase copper deposition in the brain.^11^ Rather, DPA therapy is effective to improve copper deposition in the liver and prevent liver failure progression.^2^ A study using Wilson's disease mice model reported that copper‐impaired hepatocyte function and facilitated liver cancer development through oxidative stress, and thiamine administration suppressed cancer development for its mechanisms.^12^ Therefore, DPA is useful to prevent HCC occurrence in Wilson's disease. Our case exhibited a rare complication of HCC in Wilson's disease treated with DPA for 36 years. Based on the Rhodanine staining, the remaining copper deposition in the liver might cause fatty liver change and HCC occurrence, probably due to insufficient chelation; however, another possibility arises that DPA treatment results in iron accumulation in the liver, which causes synergistic radical formation with copper and causes hepatic fibrosis and liver cancer, as previously reported.^13^ Therefore, DPA is considered useful HCC occurrence prevention^14^; however, long‐term follow‐up is essential.

## Case Presentation

**Figure 1:**
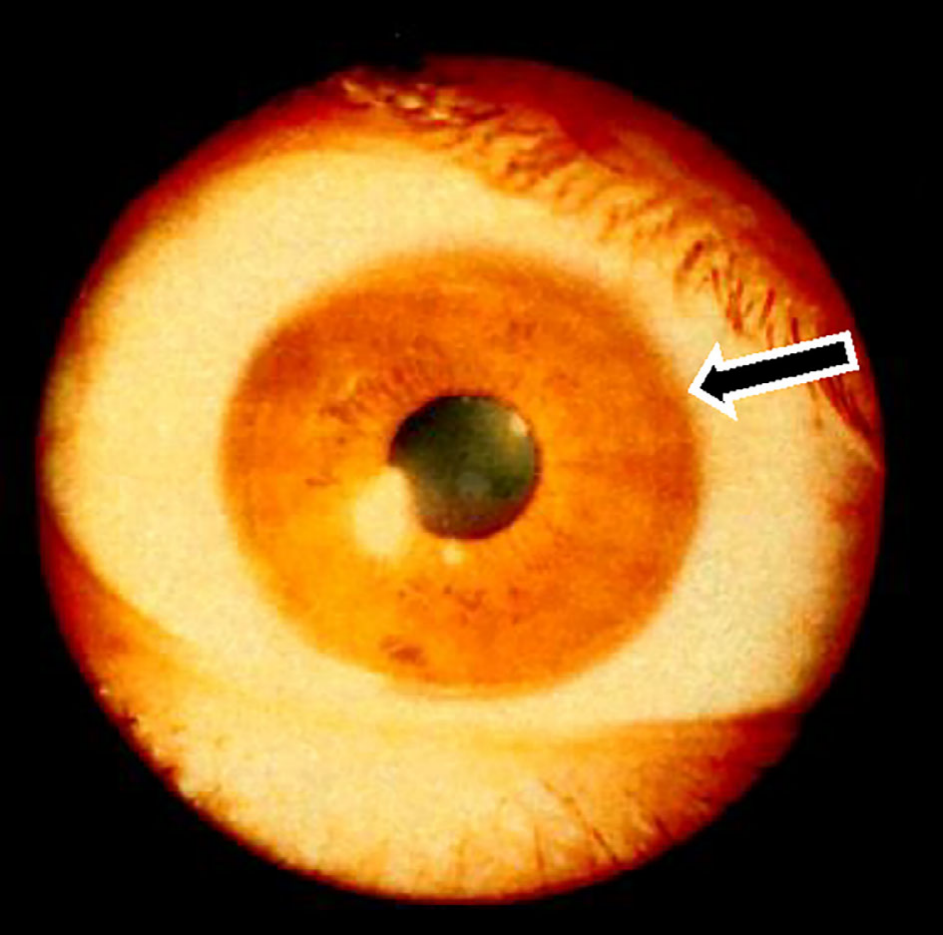
Kayser–Fleischer ring (black arrow).

**Figure 2:**
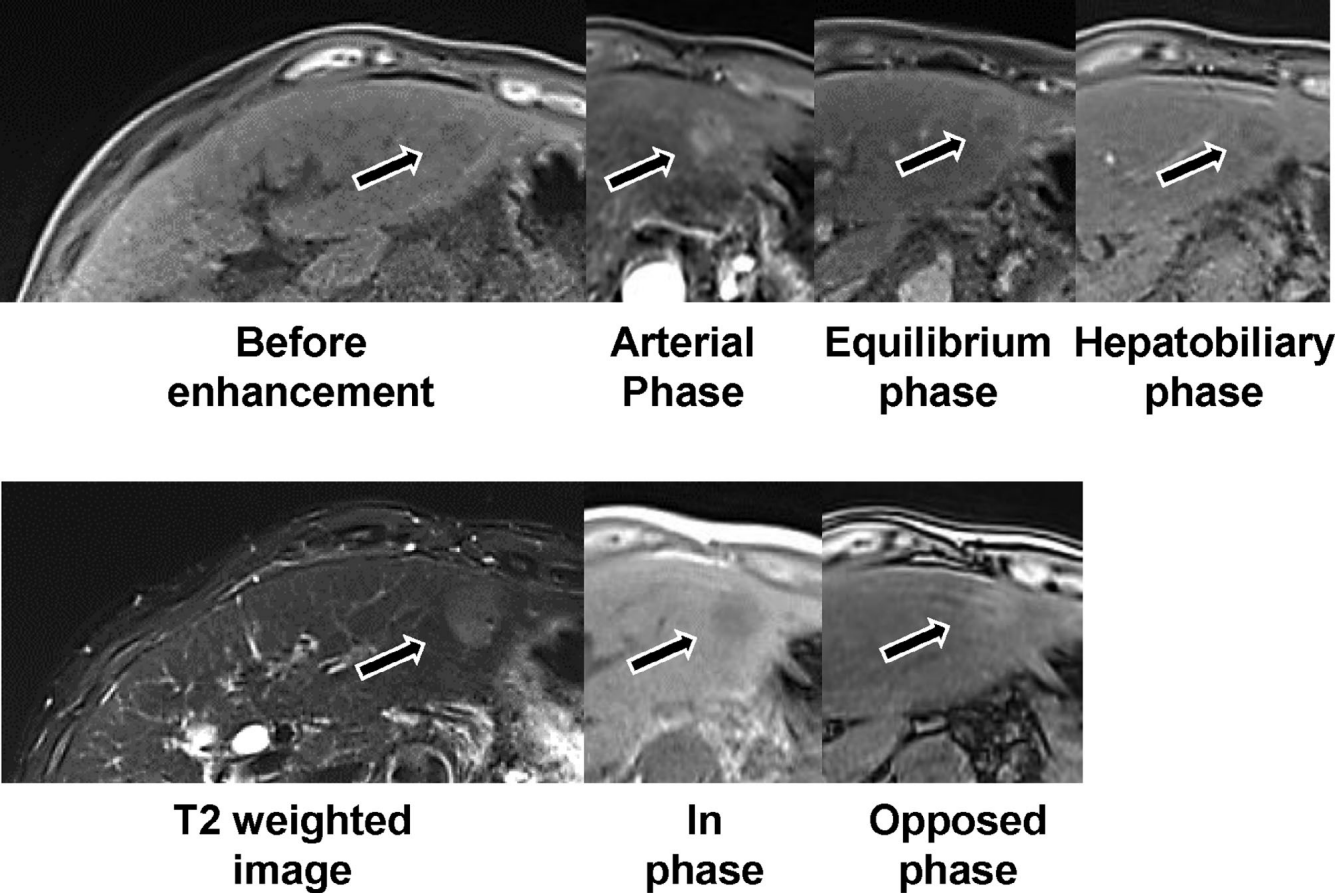
Magnetic resonance imaging (MRI) of the liver. The liver exhibited mildly dull‐edged surface and high‐intensity signal in T1 in‐phase and low in opposed phase in MRI. The tumor revealed low intensity in T1 in‐phase and high in opposed phase, high‐intensity signal in T2‐weighted image, and early enhancement effect in the arterial phase and low intensity in the hepatobiliary phase of Gadolinium‐ethoxybenzyl‐diethylenetriamine pentaacetic acid (Gd‐EOB‐DTPA) MRI (black arrows).

**Figure 3:**
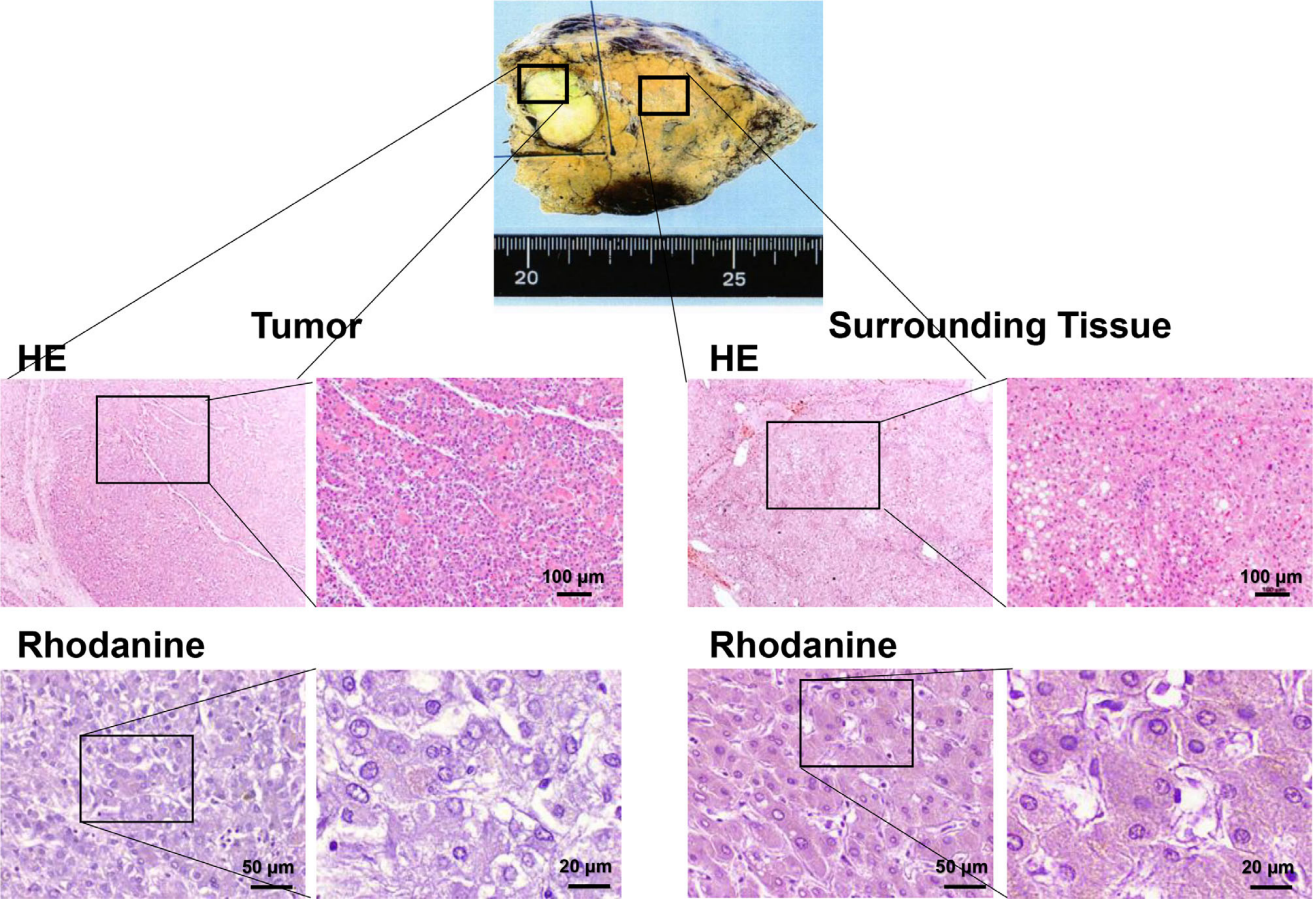
Histological analysis of tumor. A macroscopic of the tumor revealed a 2 cm whitish mass in the liver. The histological analyses revealed a moderately differentiated hepatocellular carcinoma surrounded by cirrhotic tissue with fatty infiltration (Hematoxylin and eosin staining [HE]). Rhodanine staining revealed a slight positively stained area in both tumor and surrounding tissues (black arrows).

A 62‐year‐old female who was diagnosed with Wilson's disease with Kayser–Fleischer ring (Fig. 1) and neurological symptoms treated with DPA for 36 years exhibited a mild increase of tumor markers of alpha‐fetoprotein (AFP) to 113 ng/mL upon scheduled checkup every 3 months. She had a history of hypertension, esophageal varices, and chronic glomerulonephritis, without drinking history. Her parents had a consanguineous marriage as cousins, and a sister of the patient died from impaired liver function at the age of nine. Currently, she had neurological symptoms of mild dysarthria and dyskinesia but no abdominal symptoms. Laboratory findings revealed mild thrombocytopenia of 129, 000 platelets per microliter, mild increase in AFP as described above, without other abnormal findings. The liver revealed mildly dull‐edged surface and high‐intensity signal in T1 in‐phase and low in opposed phase in magnetic resonance imaging (MRI), reflecting cirrhotic changes with fat infiltration. The tumor revealed the opposite pattern, high‐intensity signal in T2 weighted image, and early enhancement effect and low intensity in the hepatobiliary phase of Gadolinium‐ethoxybenzyl‐diethylenetriamine pentaacetic acid (Gd‐EOB‐DTPA) MRI (Fig. 2). The surrounding liver exhibited poor uptake of Gd‐EOB‐DTPA in hepatobiliary phase due to fatty infiltration. As the case revealed good hepatic function of Child–Pugh class A and general condition, the tumor was surgically resected. The tumor revealed a 2 cm mass, and histological analyses revealed a moderately differentiated HCC surrounded by fibrotic tissue diagnosed as liver cirrhosis with fatty infiltration. Rhodanine staining revealed slight positively stained area in both tumor (Fig. 3) and surrounding tissues. So far, the patient revealed no recurrence, with normal serum AFP level.
